# Association of C-reactive protein, tumor necrosis factor-alpha, and interleukin-6 with chronic kidney disease

**DOI:** 10.1186/s12882-015-0068-7

**Published:** 2015-05-30

**Authors:** Belinda T. Lee, Faheemuddin A. Ahmed, L. Lee Hamm, Federico J. Teran, Chung-Shiuan Chen, Yanxi Liu, Kamal Shah, Nader Rifai, Vecihi Batuman, Eric E. Simon, Jiang He, Jing Chen

**Affiliations:** Department of Medicine, Tulane University School of Medicine, New Orleans, LA USA; Hypertension and Renal Center of Excellence, Tulane University School of Medicine, New Orleans, LA USA; Southeast Louisiana Veterans Health Care System, New Orleans, LA USA; Department of Epidemiology, Tulane University School of Public Health and Tropical Medicine, New Orleans, LA USA; Department of Laboratory Medicine, Children’s Hospital, Harvard School of Medicine, Boston, MA USA

**Keywords:** C-reactive protein, Choric kidney disease, Inflammation, Interleukin-6, Tumor necrosis factor-alpha

## Abstract

**Background:**

We studied the association of inflammatory biomarkers including C-reactive protein (CRP), tumor necrosis factor-alpha (TNF-α), and interleukin-6 (IL-6) with chronic kidney disease (CKD).

**Methods:**

We conducted a case–control study among 201 CKD patients and 201 community-based controls in the greater New Orleans area. CKD was defined as estimated-glomerular filtration rate (eGFR) <60 mL/min/1.73 m^2^ or albuminuria ≥30 mg/24-h. Serum CRP, TNF-α, and IL-6 were measured using standard methods. Multivariable regression models were used to examine associations between the inflammatory biomarkers and CKD adjusting for important CKD risk factors, history of cardiovascular disease, and use of antihypertensive, antidiabetic, and lipid-lowering agents and aspirin.

**Results:**

The multivariable-adjusted medians (interquartile-range) were 2.91 (1.47, 5.24) mg/L in patients with CKD vs. 1.91 (0.99, 3.79) mg/L in controls without CKD (p = 0.39 for group difference) for CRP; 1.86 (1.51, 2.63) pg/mL vs. 1.26 (1.01, 1.98) pg/mL (p < 0.0001) for TNF-α; and 2.53 (1.49, 4.42) pg/mL vs. 1.39 (0.95, 2.15) pg/mL (p = 0.04) for IL-6, respectively. Compared to the lowest tertile, the highest tertile of TNF-α (OR 7.1, 95 % CI 3.2 to 15.5) and IL-6 (OR 2.5, 95 % CI 1.1 to 5.5) were significantly associated with higher odds of CKD in multivariable-adjusted models. Additionally, higher TNF-α and IL-6 were independently and significantly associated with lower eGFR and higher albuminuria.

**Conclusions:**

Our data suggest that TNF-α and IL-6, but not CRP, are associated with the prevalence and severity of CKD, independent from established CKD risk factors, history of cardiovascular disease, and use of antihypertensive, antidiabetic, and lipid-lowering agents and aspirin.

## Background

Chronic kidney disease (CKD) is highly prevalent worldwide and a major risk factor for end-stage renal disease (ESRD), cardiovascular disease (CVD), and premature death [1]. Systemic inflammation has been associated with increased risk of CVD and mortality in patients with CKD [2]. For example, C-reactive protein (CRP), an acute phase reactant, is associated with all-cause and CVD mortality in patients with ESRD [3–7]. Tumor necrosis factor-alpha (TNF-α) and interleukin-6 (IL-6), key cytokines that mediate both acute and chronic inflammation, are associated with CVD morbidity and mortality in the general population [8–10] and in predialysis and dialysis patients [11, 12]. However, several epidemiological studies have reported inconsistent findings on the associations of CRP, TNF-α, IL-6, and CKD. For instance, Gupta and colleagues reported that CRP was inversely associated with estimated glomerular filtration rate (eGFR) but not associated with urine albumin-to-creatinine ratio in the Chronic Renal Insufficiency Cohort (CRIC) Study [13]. Upadhyay and colleagues reported that multivariable-adjusted means of TNF-α and IL-6, but not CRP, were significantly elevated among patients with CKD in the Framingham Offspring cohort [14]. In addition, it remains uncertain if the association of CRP, TNF-α, and IL-6 with CKD is independent of traditional CKD risk factors, history of CVD, and use of antihypertensive, antidiabetic, and lipid-lowering agents and aspirin.

The objective of this case–control study is to investigate the association of circulating CRP, TNF-α, and IL-6 with CKD after adjusting for multiple important risk factors, including current treatment for hypertension, hyperlipidemia, diabetes, and CVD. In addition, we examine the association between circulating CRP, TNF-α, and IL-6 with CKD severity, measured by eGFR and urinary albumin excretion.

## Methods

### Study participants

We recruited 201 CKD patients and 201 controls without CKD in the greater New Orleans, Louisiana area from 2007 to 2010. CKD patients were age 21 to 74 years and recruited from nephrology and internal medicine clinics in the study area. CKD was defined as eGFR <60 ml/min/1.73 m^2^ or the presence of albuminuria (≥30 mg/24-h) for three or more months. Patients with acute kidney injury were excluded. In addition, patients were excluded if they had a history of chronic dialysis, kidney transplant, immunotherapy in the past six months, chemotherapy within the past two years, or current clinical trial participation that may have an impact on CKD. Additional exclusion criteria were history of HIV or AIDS and inability or unwillingness to give informed consent. Control participants were recruited through mass mailings to residents aged 21–74 years living in the same area according to zip code.

Tulane University Internal Review Board approved this study, and written informed consent was obtained at the screening visit from all study participants. This study adheres to the Declaration of Helsinki.

### Study measurements

A standard questionnaire was administered by trained staff at a clinical visit to obtain demographic information, lifestyle risk factors (including cigarette smoking and alcohol ingestion), self-reported history of CVD, diabetes, hypercholesterolemia, and hypertension, as well as the use of antihypertensive, lipid-lowering, and anti-diabetic medications.

Three blood pressure (BP) measurements were obtained at a clinical visit by trained and certified staff according to a common protocol adapted from procedures recommended by the American Heart Association [15]. A standard mercury sphygmomanometer was used, and one of four cuff sizes (pediatric, regular adult, large, or thigh) was chosen on the basis of the circumference of the participant’s arm. Body height and weight were obtained by trained staff and used to calculate body mass index (BMI = weight in kg/height^2^ in m) and body surface area using Mosteller’s formula [(weight in kg × height in cm)/3600]^1/2^ [16].

An overnight fasting blood sample was collected to measure plasma glucose, serum creatinine, cholesterol, and triglycerides. A 24-h urinary sample was collected to measure creatinine and albumin. Serum cholesterol and triglyceride levels were assayed using an enzymatic procedure on the Hitachi 902 automatic analyzer (Roche Diagnostics, Indianapolis, IN, USA). Serum glucose was measured using a hexokinase enzymatic method (Roche Diagnostics, Indianapolis, IN, USA). Serum creatinine was measured using the Roche enzymatic method (Roche–Hitachi P-Module instrument with Roche Creatininase Plus assay, Hoffman-La Roche, Basel, Switzerland). Urinary concentrations of albumin and creatinine were measured with a DCA 2000 Analyzer (Bayer AG, Leverkusen, Germany). eGFR was estimated from serum creatinine, sex, age, and race using the CKD-Epi equation [17].

Serum levels of CRP were measured by a high-sensitivity latex-enhanced immunonephelometric assay (Roche Diagnostics, Indianapolis, IN). The assay has a sensitivity of 0.02 mg/L and an intra-assay coefficient of variation (CV) of <1.0 % and an inter-assay CV of 3.7 %. Serum levels of IL-6 were measured by a commercially available ELISA (Quantikine HS human IL-6 Immunoassay kit, R&D System, Minneapolis, MN). The detectable limit for IL-6 is 0.039 pg/mL, the intra-assay CV is from 6.9 % to 7.8 %, and the inter-assay CV is from 6.5 % to 9.6 %. Serum TNF-α levels were measured by a commercially available ELISA (Quantikine HS human TNF-α Immunoassay kit, R & D Systems, Minneapolis, MN). The detectable limit for TNF-α is 0.12 pg/mL, the intra-assay CV is from 5.3 % to 8.8 %, and the inter-assay CV is from 10.8 % to 16.7 %.

### Statistical analysis

Medians and interquartile ranges for serum CRP, IL-6, and TNF-α were calculated for CKD patients and controls, and the Mann–Whitney U test was used to test differences in the unadjusted medians. Quantile regression was used to obtain adjusted medians (interquartile ranges) and the Wald test was used to test differences in the adjusted medians between CKD patients and controls [18]. These analyses were adjusted for age, gender, race, high-school education, physical activity, current cigarette smoking, weekly alcohol drinking, body-mass index, LDL-cholesterol, plasma glucose, systolic blood pressure, history of CVD, use of antihypertensive, anti-diabetic, and lipid-lowering agents, and aspirin.

Multivariate logistic regression was used to obtain adjusted odds ratios comparing the higher tertiles of CRP, IL-6, and TNF-α levels to the lowest tertile between CKD patients and controls. Serum tertile levels were defined based upon measurements in the control group. In addition, multivariable linear regression was used to examine the association of serum levels of CRP, IL-6, and TNF-α with eGFR and albuminuria after adjustment for the previously mentioned covariates. Log transformations were used for CRP, IL-6, TNF-α, and urinary albumin levels, because they were not normally distributed. We did not impute missing data. All analyses were performed using SAS version 9.2 statistical software (Cary, NC).

Our study was designed to have an 80 % statistical power to detect an odds ratio of 1.77 at a 2-sided significance level of 0.05 when the cases and controls were compared by the tertile of biomarkers. In addition, we have an 80 % statistical power to detect a mean difference of 0.3 standard deviations of biomarkers between the cases and controls at a 2-sided significance level of 0.05.

## Results

The general characteristics of study participants by CKD status are presented in Table 1. Patients with CKD had a mean eGFR of 43.4 mL/min/1.73 m^2^ compared to 96.7 mL/min/1.73 m^2^ among controls without CKD. Among 201 CKD patients, 5 were stage-1, 33 stage-2, 104 stage-3, 49 stage-4, and 10 stage-5. Median urinary albumin excretion was 74.5 mg/24 h in CKD patients and 5.9 mg/24 h in controls. Those with CKD were older, less educated, and less likely to drink alcohol or engage in regular physical activity compared to those without CKD. In addition, they were more likely to have a history of CVD, hypertension, diabetes, and hypercholesterolemia. Mean BMI, systolic BP, and serum glucose levels were significantly higher, while LDL- and HDL-cholesterol were lower in CKD patients compared to controls.

**Table 1:** Characteristics of the Participants by Chronic Kidney Disease Status

Variables	CKD patients (n = 201)	Non-CKD controls (n = 201)	*P*-value for difference
Age, yrs	55.9 ± 9.9	52.5 ± 10.0	0.0007
Male, %	55.2	45.3	0.05
Blacks, %	60.7	51.2	0.06
High school education, %	58.5	81.6	<0.0001
Current cigarette smoking, %	53.7	48.8	0.32
Weekly alcohol drinking, %	27.9	59.2	<0.0001
Regular physical activity^a^, %	53.0	72.9	<0.0001
History of CVD, %	43.7	7.0	<0.0001
History of hypertension, %	88.1	23.9	<0.0001
History of diabetes, %	49.3	5.5	<0.0001
History of hypercholesterolemia, %	65.7	30.9	<0.0001
Body-mass index, kg/m^2^	32.2 ± 7.8	28.9 ± 6.4	<0.0001
Systolic blood pressure, mmHg	132.2 ± 21.0	122.0 ± 14.7	<0.0001
Diastolic blood pressure, mmHg	77.2 ± 13.5	77.6 ± 9.4	0.77
Plasma glucose, mg/dL	119.9 ± 46.7	103.4 ± 35.4	<0.0001
LDL-cholesterol, mg/dL	103.3 ± 45.9	118.2 ± 30.2	<0.0001
HDL-cholesterol, mg/dL	50.3 ± 15.6	57.7 ± 18.0	<0.0001
eGFR, mL/min/1.73 m^2^	43.3 ± 19.9	96.7 ± 16.8	<0.0001
Urinary albumin, mg/24-h	74.5 (12.3, 417.4)	5.9 (4.1, 11.4)	<0.0001

Mean ± standard deviation, percentage, or median (inter-quartile range)

*LDL* low-density lipoprotein; *HDL* high-density lipoprotein; *eGFR* estimated-glomerular filtration rate; and *CVD* cardiovascular disease

^a^Moderate or heavy physical activity at least twice per week

The median serum levels of CRP, IL-6, and TNF-α were significantly higher in patients with CKD compared to controls. After adjusting for important covariables, the medians of IL-6 and TNF-a, but not CRP, remained significantly higher (Table 2).

**Table 2:** Medians and inter-quartile range of inflammatory biomarkers according to chronic kidney disease status

Inflammatory Biomarkers	Unadjusted median (IQR)			Multivariable-adjusted median (IQR)^a^		
	CKD patients (n = 201)	Non-CKD controls (n = 201)	*P*-value for difference	CKD patients (n = 201)	Non-CKD controls (n = 201)	*P*-value for difference
CRP, mg/L (IQR)	2.80 (1.13, 5.61)	1.49 (0.75, 3.65)	<0.0001	2.91 (1.47, 5.24)	1.91 (0.99, 3.79)	0.39
TNF-α, pg/mL (IQR)	1.82 (1.5, 2.51)	1.27 (0.99, 1.81)	<0.0001	1.86 (1.51, 2.63)	1.26 (1.01, 1.98)	<0.0001
IL-6, pg/mL (IQR)	2.46 (1.32, 4.54)	1.24 (0.88, 2.2)	<0.0001	2.53 (1.49, 4.42)	1.39 (0.95, 2.15)	0.04

IQR = inter-quartile range; CRP = C-reactive protein; TNF-α = tumor necrosis factor alpha; IL-6 = interleukin 6

^a^Adjusted for age, gender, race, high-school education, physical activity, current cigarette smoking, weekly alcohol drinking, body-mass index, LDL-cholesterol, plasma glucose, systolic blood pressure, history of cardiovascular disease, antihypertensive treatment, antidiabetic treatment, lipid lowering treatment, and aspirin use

The multivariate-adjusted odds ratios (95 % confidence intervals) of CKD comparing the higher two tertiles to the lowest tertile of the serum inflammatory biomarker levels from the multivariate-adjusted logistic regression analyses are presented in Table 3. The higher levels of TNF-α and IL-6, but not CRP, were significantly associated with odds of CKD after adjusting for multiple confounding factors.

**Table 3:** Odds ratios of chronic kidney disease associated with the higher tertile compared to the lowest tertiles of inflammatory biomarkers

	Age, gender, and race-adjusted		Multivariable-adjusted^a^	
	OR (95 % CI)	*P*-value for trend	OR (95 % CI)	*P*-value for trend
CRP ( mg/L)				
≤1.1	1.0 (ref)	0.0002	1.0 (ref)	0.15
>1.1-3.3	1.3 (0.8-2.2)		1.2 (0.6-2.5)	
>3.3	2.7 (1.6-4.1)		1.8 (0.8-3.9)	
TNF-α (ng/mL)				
≤1.3	1.0 (ref)	<0.0001	1.0 (ref)	<0.0001
>1.3-1.9	6.6 (3.7-11.7)		5.8 (2.7-12.7)	
>1.9	7.8 (4.4-13.8)		7.1 (3.2-15.5)	
IL-6 (μg/mL)				
≤1.2	1.0 (ref)	<0.0001	1.0 (ref)	0.02
>1.2-2.6	1.7 (1-2.8)		1.1 (0.5-2.3)	
>2.6	5.0 (2.8-8.6)		2.5 (1.1-5.5)	

*CI* confidence interval; *CRP C*-reactive protein; *TNF-α* tumor necrosis factor alpha; *IL-6* interleukin 6

^a^Adjusted for age, gender, race, high-school education, physical activity, current cigarette smoking, weekly alcohol drinking, body-mass index, LDL-cholesterol, plasma glucose, systolic blood pressure, history of cardiovascular disease, antihypertensive treatment, antidiabetic treatment, lipid lowering treatment, and aspirin use

In multivariable-adjusted linear regression analyses, serum levels of TNF-α and IL-6 were inversely and significantly related to eGFR and positively related to urinary albumin excretion (Table 4). On the other hand, serum CRP level was not significantly associated with either eGFR or urine albumin excretion.

**Table 4:** Multivariable-adjusted regression coefficients (95 % confidence intervals) of estimated-glomerular filtration rate and urinary albumin associated with a one standard deviation difference in inflammatory biomarkers

Inflammatory biomarkers (SD)	Estimated-glomerular filtration rate, mL/min/1.73 m^2^		Log (urinary albumin, mg/24-h)	
	β (95 % CI)	*P*-value	β (95 % CI)	*P*-value
CRP (Log,1.33 mg/L)	−1.27 (−4.11 to 1.58)	0.38	0.11 (−0.08 to 0.29)	0.27
TNF-α (Log,0.69 ng/mL)	−5.02 (−7.56 to −2.47)	0.0001	0.27 (0.1 to 0.44)	0.002
IL-6 ( Log, 0.9 ng/mL)	−4.69 (−7.65 to −1.73)	0.0028	0.35 (0.16 to 0.54)	0.0003

Adjusted for age, gender, race, high-school education, physical activity, current cigarette smoking, weekly alcohol drinking, body mass index, LDL-cholesterol, plasma glucose, systolic blood pressure, history of cardiovascular disease, antihypertensive treatment, antidiabetic treatment, lipid lowering treatment, and aspirin use

*SD* standard deviation; *log* log-transformation; *CI* confidence interval; *CRP* C-reactive protein; *TNF-α* tumor necrosis factor alpha; *IL-6* interleukin 6

## Discussion

The present study shows that inflammatory biomarkers TNF-α and IL-6 are significantly elevated in patients with CKD compared to controls without CKD. In addition, TNF-α and IL-6 are significantly and positively associated with the severity of CKD, measured by eGFR and urinary albumin excretion. These associations are independent of established risk factors for CKD, history of CVD, and use of antihypertensive, antidiabetic, and lipid-lowering agents, as well as aspirin. In this study, serum level of CRP is not independently associated with CKD.

CRP is a well-recognized risk factor for CVD and mortality in the general population as well as in patients with ESRD [3–7, 19]. However, our study indicates that circulating CRP level was not significantly elevated in pre-dialysis CKD patients after accounting for established CKD risk factors, history of CVD, and use of antihypertensive, antidiabetic, and lipid-lowering agents, and aspirin. This finding is consistent with several previous reports [14, 20–22]. In addition, our study reports that serum CRP was not associated with eGFR or albuminuria in multiple linear regression models. In contrast, a previous cross-sectional epidemiological study suggested that CRP was associated with eGFR but not urine albumin-to-creatinine ratio among patients with CKD [13]. However, two longitudinal epidemiologic studies also suggested that CRP was not associated with the risk of developing CKD [23, 24]. Taken together, these findings suggest that CRP is not an independent risk factor for CKD.

Our study shows that circulating TNF-α and IL-6 levels are significantly higher in CKD patients compared to controls and the levels of TNF-α and IL-6 are independently associated with the severity of CKD. These findings are consistent with those of previous large epidemiological studies [13, 14]. The longitudinal study by Shankar *et al.* also suggested that TNF-α receptor 2 and IL-6 levels were positively associated with the risk of developing CKD [23]. However, it is possible that albuminuria selectively activates cytokines and elevates TNF-α and IL-6 levels. In addition, there may be decreased clearance of these cytokines in patients with CKD [25]. Therefore, future studies should investigate whether the relationships of TNF-α and IL-6 with proteinuria are causal.

The current study is noteworthy because it reports that TNF-α and IL-6, but not CRP, are significantly associated with the prevalence and severity of CKD, independent of established risk factors, history of CVD, and use of antihypertensive, antidiabetic, and lipid-lowering agents, as well as aspirin. Careful measures of study exposure and outcome variables in this study allowed for precise estimation of the association. In addition, controls were selected from the community, which should reduce potential selection bias. Furthermore, the study participants included a racially diverse group with variable degrees of renal function and urine albumin excretion. Potential limitations of this study should be noted. First, this study cannot establish a temporal relationship or make causality inferences due to its cross-sectional, observational nature. Second, the study included a single measurement of the proinflammatory cytokines, which may not represent average levels of these biomarkers over time. However, patients with acute kidney injury were excluded from the present study. In addition, serum creatinine-based eGFR was used to define kidney function in our study. The use of cystatin C-based or cystatin- and creatinine-based eGFR might provide a better assessment of kidney function regarding its predictive value for the risks of death and ESRD [26]. Finally, we were not able to analyze the association between inflammatory markers and subtype of CKD by different causes because renal biopsy data were not available in our study.

## Conclusions

Our study findings suggest that TNF-α and IL-6, but not CRP, are associated with the prevalence and severity of CKD independent of established CKD risk factors, history of CVD, and use of antihypertensive, antidiabetic, and lipid-lowering agents and aspirin. Further studies are warranted to investigate the role of inflammatory biomarkers in the etiology of CKD and their predictive value for advanced CVD and ESRD events among patients with CKD.

## Abbreviations

BP: Blood pressure
BMI: Body-mass index
CKD: Chronic kidney disease
CRP: C-reactive protein
CV: Coefficient of variation
CVD: Cardiovascular disease
eGFR: Estimated glomerular filtration rate
ESRD: End-stage renal disease
IL-6: Interleukin-6
TNF-α: Tumor necrosis factor-alpha
